# Secure Asynchronous Communication Between Smokers and Tobacco Treatment Specialists: Secondary Analysis of a Web-Assisted Tobacco Intervention in the QUIT-PRIMO and National Dental PBRN Networks

**DOI:** 10.2196/13289

**Published:** 2020-05-06

**Authors:** Rajani Shankar Sadasivam, Ariana Kamberi, Kathryn DeLaughter, Barrett Phillips, Jessica H Williams, Sarah L Cutrona, Midge N Ray, Gregg H Gilbert, Thomas K Houston, QUITPRIMO, National Dental PBRN Collaborative Group

**Affiliations:** 1 University of Massachusetts Medical School Worcester, MA United States; 2 Edith Nourse Rogers Memorial Veterans Hospital Bedford, MA United States; 3 Veterans Affairs Central Western Massachusetts Healthcare System Leeds, MA United States; 4 University of Alabama at Birmingham Birmingham, AL United States

**Keywords:** distance counseling, tobacco cessation, internet-based intervention, smoking cessation

## Abstract

**Background:**

Within a web-assisted tobacco intervention, we provided a function for smokers to asynchronously communicate with a trained tobacco treatment specialist (TTS). Previous studies have not attempted to isolate the effect of asynchronous counseling on smoking cessation.

**Objective:**

This study aimed to conduct a semiquantitative analysis of TTS-smoker communication and evaluate its association with smoking cessation.

**Methods:**

We conducted a secondary analysis of data on secure asynchronous communication between trained TTSs and a cohort of smokers during a 6-month period. Smokers were able to select their preferred TTS and message them using a secure web-based form. To evaluate whether the TTS used evidence-based practices, we coded messages using the Motivational Interviewing Self-Evaluation Checklist and Smoking Cessation Counseling (SCC) Scale. We assessed the content of messages initiated by the smokers by creating topical content codes. At 6 months, we assessed the association between smoking cessation and the amount of TTS use and created a multivariable model adjusting for demographic characteristics and smoking characteristics at baseline.

**Results:**

Of the 725 smokers offered asynchronous counseling support, 33.8% (245/725) messaged the TTS at least once. A total of 1082 messages (TTSs: 565; smokers 517) were exchanged between the smokers and TTSs. The majority of motivational interviewing codes were those that supported client strengths (280/517, 54.1%) and promoted engagement (280/517, 54.1%). SCC code analysis showed that the TTS provided assistance to smokers if they were willing to quit (247/517, 47.8%) and helped smokers prepare to quit (206/517, 39.8%) and anticipate barriers (197/517, 38.1%). The majority of smokers’ messages discussed motivations to quit (234/565, 41.4%) and current and past treatments (talking about their previous use of nicotine replacement therapy and medications; 201/565, 35.6%). The majority of TTS messages used behavioral strategies (233/517, 45.1%), offered advice on treatments (189/517, 36.5%), and highlighted motivations to quit (171/517, 33.1%). There was no association between the amount of TTS use and cessation. In the multivariable model, after adjusting for gender, age, race, education, readiness at baseline, number of cigarettes smoked per day at baseline, and the selected TTS, smokers messaging the TTS one or two times had a smoking cessation odds ratio (OR) of 0.8 (95% CI 0.4-1.4), and those that messaged the TTS more than two times had a smoking cessation OR of 1.0 (95% CI 0.4-2.3).

**Conclusions:**

Our study demonstrated the feasibility of using asynchronous counseling to deliver evidence-based counseling. Low participant engagement or a lack of power could be potential explanations for the nonassociation with smoking cessation. Future trials should explore approaches to increase participant engagement and test asynchronous counseling in combination with other approaches for improving the rates of smoking cessation.

## Introduction

### Background

Smoking is the number one preventable cause of death [1]. Nearly 1 in 5 adults in the United States, or 42.1 million people, currently smoke cigarettes [2]. Quitting reduces the risk of smoking-related diseases, including cardiovascular diseases [3]. Current guidelines recommend that smokers use behavioral counseling to aid their quitting efforts [4]. However, most smokers do not use counseling in their quit attempts. For instance, despite the wide availability of Quitlines, a telephone-based tobacco counseling that has been established in all 50 states, it has been reported that only 1% to 2% of smokers use Quitline services [5,6]. More innovations are needed to increase access to counseling resources [1].

Providing behavioral counseling by trained tobacco treatment specialists (TTSs) over the internet may help overcome barriers to access. Internet access is almost ubiquitous in the United States, especially with the increasing use of mobile phones [7-9]. Users have also become comfortable communicating over the web (eg, emails, text messages, and on social networks). Web-based counseling is usually delivered synchronously through live chat, where the counselor and smoker are both online and engage via video, audio, or messaging. Synchronous counseling has the benefit of the smokers being able to directly interact with the counselors, and for the counselors to be able to tailor the counseling to the smoker. Previous papers have evaluated synchronous counseling [10-12]. However, synchronous counseling may not be convenient for all smokers as they need to coordinate their schedules with the counselors. Asynchronous counseling that allows the smokers the convenience of communicating with the counselor at any time can be used to augment synchronous counseling. Asynchronous counseling can be achieved using a secure Health Insurance Portability and Accountability Act–compliant form of email or secure messaging. Previous studies have provided functions to communicate asynchronously with experts such as a question and answer email service or a text messaging interface for communicating with a human coach [13-15]. These functions have mostly been provided within the context of a larger intervention. To our knowledge, none of these studies conducted secondary analysis to understand and explore the effect of these counseling efforts.

### Objective

Our objective is to conduct a secondary evaluation of asynchronous web-based counseling provided by trained TTSs to smokers participating in the QUIT-PRIMO randomized trial testing a web-assisted tobacco intervention (WATI) [16,17]. As published before [16-18] and further described below, in the QUIT-PRIMO randomized trial, the recruited smokers were randomized to the following three groups: a WATI control (control), the WATI enhanced with proactive, pushed tailored email motivational messaging (messaging), and the WATI with messaging further enhanced with personal secure messaging with a TTS (personalized). Although we found significant differences between those who received the motivational messages (personalized and messaging groups) versus the control (17%; odds ratio [OR] 1.69; 95% CI 1.03-2.8; *P*=.04), we found no differences between the personalized and the messaging group of the study. 

Understanding TTS use by the smokers might provide us more insight into why the access to TTS did not result in an increase in smoking cessation in our study and may help in designing more patient-centered asynchronous counseling support in future web-based interventions. In this paper, we add to this literature by conducting a semiquantitative analysis of the TTS-smoker communication and evaluating its association with smoking cessation. We report the frequency of TTS-smoker communication, whether the TTSs were able to incorporate evidence-based practices, such as motivational interviewing, into their counseling. Our analysis also included the communication initiated by the smokers.

## Methods

### Study Design, Setting, and Sample

We conducted a longitudinal, observational cohort study of secure asynchronous communication between 3 trained TTSs and 245 smokers. The communication occurred during a 6-month period [16]. For the purpose of this study, we only included smokers from the personalized group. These smokers were able to select from among 3 TTSs and initiate the communication by messaging their TTS using a secure web-based form. The TTSs were, then, able to message back using an administrative portal. We implemented the *secure* communication similar to how it is implemented on web-based patient portals. When someone initiated a contact through the website, an email alert was sent to the smoker and the TTS. This email alert did not contain any personal identifiers and the smoker and the TTS had to log in to the website to view their messages. Our website was available only through an encrypted channel using the hypertext transfer protocol secure.

The approach for selecting and messaging the TTS and coding messages is described in detail below (Choosing a Tobacco Treatment Specialist in the Personalized Group). Our study was approved by the University of Alabama at Birmingham and the University of Massachusetts Medical School Institutional Review Boards.

### The QUIT-PRIMO Randomized Trial

#### Recruitment

Smokers were recruited during June 2010-March 2012. Smoker recruitment was conducted through medical and dental practice patient referrals as well as through Google advertisements [16-19]. Our recruitment process has been described in detail in our previous publications. Briefly, primary care medical practices were recruited from a registered database of internal medicine and family or general practitioners. Dental practices were recruited from state lists of registered dentists and through the Dental Practice–Based Research Network. At these practices, we implemented an electronic referral program that allowed providers (doctors, nurses, or other clinical staff) to recruit smokers to the Decide2Quit.org WATI at the point of care by entering their email addresses into a web-based form. When smokers were electronically referred, they were sent up to 10 email messages encouraging registration over an 8-week period or until the patient registered. To participate on Decide2Quit.org, all smokers completed a web-based consent and registered on the website. To recruit smokers through Google advertisements, 3 advertisements were posted on Google AdWords. Advertisements were linked to searches for keywords related to smoking (eg, smoking, quit smoking, stop smoking, quit, quit smoking tips, and quit smoking programs) and included a link that took participants directly to the Decide2Quit.org home page where they could choose to register as new participants. Each smoker had access to Decide2quit.org for 6 months.

#### Design of the Randomized Trial

The Decide2Quit.org WATI was designed for all smokers, supporting cessation induction for those not ready to quit and acting as an aid to cessation for those preparing to quit [16-19]. Decide2Quit.org was implemented as an adaptable service with modules that could be engaged based on assigned group. Thus, smokers were allocated to one of three increasingly enhanced versions of Decide2Quit.org. The interventions received by the three randomized groups of smokers are described below.

#### The Control Group

Smokers randomized to the active control received *an interactive, tailored quit smoking website*. This module included motivational information tailored to readiness to quit (not thinking of quitting, thinking of quitting, and preparing to quit), and interactive risk, decisional balance, and cessation barrier calculators, and games linking the chemicals in smoking with their other uses (eg, formaldehyde is used in both cigarettes and in embalming). The control also included a library of informational resources about smoking, and sections on seeking social support and talking to your doctor about quitting. These tools are variations of those in many previously studied evidence-based WATI.

#### The Messaging Group

##### Pushed Motivational Email Messages Module Plus Control Module

For this group, we enhanced the control with a pushed motivational email messaging system. Brief motivational email messages were further tailored to an individual smoker’s readiness to quit (not ready to quit, thinking about quitting, preparing to quit, and actively quitting). In the first week of registration, 4 email messages were sent to the smoker, followed thereafter by 2 email messages per week. To enhance the personal relevance of messages, our motivational email messaging system included messages written by smokers for other smokers [20].

##### Personalized Group: Personal Support Module, Messages Module, and Control Module

In addition to the above functions, the personal support module further included an innovative secure messaging portal allowing asynchronous electronic communication between smokers and trained TTSs hired to participate in the intervention team. Further details on how the patient chose and communicated with the TTS are provided below.

### Choosing a Tobacco Treatment Specialist in the Personalized Group

During registration over the web, smokers were asked to select between 3 trained TTSs. Smokers were presented the counseling philosophies of the 3 trained TTSs to facilitate this selection. The 3 TTSs created their philosophies from personal experiences and accredited TTS training [21]. Of the 3 TTSs, 2 were trained at the University of Mississippi, Medical Center ACT, while 1 was trained at the University of Massachusetts, Center for Tobacco Treatment Research and Training. Both training programs were accredited by the Council for Tobacco Treatment Training Programs **[22,23].** Content of this training consisted of bio, psycho, and social determinants of nicotine dependence, pharmacotherapy, and counseling theory and practice, which included motivational interviewing techniques, treatment strategies, and program and system issues.

Counseling philosophies of the 3 TTSs were as follows:

It is not about the quantity of your life, it is about the quality. Smoking harms nearly every organ in the body and one out of every two smokers will die prematurely from a smoking-related disease. When you think about it, those are not very good odds. Many of my loved ones struggle with tobacco use so I know first-hand how difficult quitting can be. This is why I take a holistic approach to helping you quit tobacco. It is important for you to understand ALL the reasons why you smoke and for you to think about strategies that will work best for you—I want to help you do that. Quitting tobacco may be one of the most difficult things you will do for yourself but is also one of the most important. Time is ticking...what are you waiting for?

TTS 1

Being healthy and happy is about learning what’s important to you and what you are willing to do to make that happen. Quitting smoking is no different. For some it’s as simple as wanting more time with their children or grandchildren, for others perhaps it’s about saving money to go on a trip. Either way, small steps now can make a big difference in years to come. For me, I like to help people look at why they want to quit and the reasons they smoke to figure out the best plan for them. Each person is unique and will have their own reasons for quitting smoking. By learning what motivates you and why you do certain things, you are better prepared to make healthy lifestyle changes. This way the skills learned from quitting smoking can be used in other areas of life.

TTS 2

Quitting smoking can truly be one of the biggest challenges you may face...As a prior smoker, I know first-hand the hold nicotine, a highly addictive drug, can have on one’s life. Quitting enabled me to see how much life I was missing. I would like to help you quit. Whether you are ready to quit today or just barely considering quitting, there are many things we can talk about! It is important for each smoker to know their own motivations. One very important motivation is to quit for those around you. Lead by example; kids of parents who quit while they are young are 5 times less likely to smoke themselves, and kids with parents who successfully quit are three times as likely to successfully quit themselves. I can help you to stop being “the smoker”; get rid of the shadow and join the ranks of those proud to say they are “smoke-free.” Choosing to quit today can mean a new life tomorrow.

TTS 3

Smokers were given the option to send a message at the time of registration or proceed to the website and send a message later. After login, smokers were presented with a web form (similar to an email form on a website) that included a textbox for writing the subject and another for writing the message to TTS (Multimedia Appendix 1). Please note that we did not require smokers to send messages. Once they submitted the message, an email was sent to the participant confirming their submission and indicating that the TTS will respond in 2 business days. The TTSs logged into the administrative portal daily to view any messages they had received. Each message on the administrative portal listed the assigned TTS. Messages were typically replied to by the TTSs within 2 business days. Once the TTS responded, the smokers were sent an email notifying that the TTS had responded to their message. Smokers had to login to the system to read the message. We designed the display of the message to resemble an email inbox. New messages were indicated by the subject in bold font and the smoker had to click on the message subject to read the response.

### Coding Process

To code messages and collect codes by categories, we used MAXQDA 10 (VERBI Software) [24-26], a qualitative data analysis software. This software facilitates coding by creating a hierarchical coding dictionary that can be directly mapped to the messages within the software. Authors AK, KL, BP, JW, HC, and SC were all involved in coding the messages. The principal investigator TH guided the process and trained the initial set of coders (BP and AK). BP participated in a training offered by the MAXQDA company on the tool and trained the rest of the coders on the tool. They also created a documentation of codes and coded an initial set of 10 messages under the guidance of TH. Each additional coder was integrated using the following process: They were provided the documentation of codes and asked to code the same 10 messages that had originally been coded by the first two coders (BP and AK). We, then, compared the new and original codes for interrater reliability (IRR). We reviewed the IRR results and compared them with the coding completed by the original coders. On the basis of this comparison and discussion, changes were made to code definitions. Each message was coded by at least 2 coders, and we calculated IRR for each set of 100 messages. After discussion, coder pairs reached agreement on codes used and resolved any discrepancies during the coding process. In the end, 100% agreement was achieved by consensus.

#### Development of Coding Schema for Analyzing the Tobacco Treatment Specialist Messages

Our coding schema was developed using the Motivational Interviewing Self-Evaluation Checklist and the Smoking Cessation Counseling (SCC) Scale. Motivational interviewing is a directive**,** patient-centered counseling style for eliciting behavior change by helping people to explore, clarify, and resolve ambivalence [27]. The Motivational Interviewing Self-Evaluation Checklist is a 7-item checklist, used as a self-evaluation tool to improve motivational interviewing skills [28-32]. This checklist is focused on engagement, assessing motivation, addressing ambivalence, promoting internal motivation, eliciting change talk, rolling with resistance, and supporting client strength. The SCC Scale is a 24-item questionnaire based on a 4-level response format. It was originally developed for nurses by the US Department of Health and Human Services to help in assessing, improving, or testing evidence-based methodologies for SCC [33]. The scale has both clinical relevance and research applications and can be used clinically to assess the quality of smoking cessation services [33,34]. We revised the SCC by converting its 4-level response to a 2-level response format, and by using the 17 items that focused on advanced and basic counseling after revising the scale.

We also assessed the content of messages initiated by the smokers. For this purpose, we iteratively developed a topical content coding schema (Multimedia Appendix 2). To develop this coding system, we first reviewed 20 messages for both TTSs and smokers and developed a preliminary coding schema based on these. We used this schema to code 20 additional messages, and then refined and added new codes if needed. We proceeded in blocks of 20 messages, and code definitions were revised several times before we finalized our content code category. The content themes included website content; treatment questions: general advice; treatment questions: advice behavioral; treatment questions: advice on treatments and over the counter medications (Rx and OTC); motivations; sociocultural; health; and current or past treatment and feedback from patients.

### Data Collection

Data were collected online on the WATI. During registration, we collected demographic and smoking characteristics, and readiness to quit. The TTS and smoker messages were also recorded in the database. At follow-up, the 30-day point prevalence smoking cessation rate was assessed as self-report using the question *Did you smoke any cigarettes during the past 30 days?* [35]. We also assessed on a Likert scale whether communicating with the TTS was helpful to the smoker.

### Data Analysis

Our analysis was conducted using STATA version 12 (StataCorp). All analyses were two-sided and alpha error was set at .05. We compared demographic characteristics and smoking behavior between those participants who had sent at least one message to the TTS and those who did not using the chi-square test or the Mantel-Haenszel method (MH odds) for trend. We used descriptive statistics (means or medians) to assess the codes in the TTS messages (Motivational Interviewing Self-Evaluation Checklist and SCC Scale codes). We compared the topical content codes in the TTS messages to the codes in the smoker messages. At the smoker level, we assessed the association between 6-month cessation and the frequency of each code in the TTS messages received by the smoker using separate logistic regression models for each code. For the independent variable for this analysis, we used the number of times the code appeared in messages to best represent the variations in TTS-smoker communication between the smokers. We assessed the association between smoking cessation and the selected TTS by adjusting for demographic characteristics and smoking characteristics at baseline. Furthermore, we assessed the association between 6-month cessation and the amount of TTS use and created a multivariable logistic model; adjusting for demographic characteristics, smoking characteristics at baseline, and the selected TTS. For this analysis, our dependent variable was the 30-day point prevalence cessation rate as described above. All those with missing follow-up data were coded as continued smoking. The amount of TTS use was the total number of messages sent to the TTS by each smoker. For our analysis, we categorized the amount of TTS use as follows: did not message the TTS, messaged the TTS 1 or 2 times, and messaged the TTS more than 2 times. We implemented a selection model using inverse probability weighting to determine the potential effect of the missing data [36,37]. First, based on covariates available within the dataset, we developed a logistic regression model to predict the amount of missing data. Then, we calculated the inverse probability of not being missing and weighted the main analysis by this probability.

## Results

### Comparing Characteristics of Smokers Using a Tobacco Treatment Specialist and Those Who Did Not Use a Tobacco Treatment Specialist

Approximately, a third of smokers (245/725, 33.8%) who were offered asynchronous counseling support sent messages at least once to the TTS (see the example of communication between smokers and TTSs in Multimedia Appendix 3). Those smokers who allowed smoking at home and who had visited a WATI before this study were more likely to use the TTS (*P*=.04 for both; Table 1). Among those who used the TTS and completed follow-up, 71% (32/45) reported that the communication was very helpful or somewhat helpful. In all, 29% (13/45) indicated that the communication was not very helpful or not at all helpful.

**Table 1:** Demographics and smoking behavior of participants, comparing those who messaged the tobacco treatment specialist (TTS) at least once and those who did not.

Patient characteristics		Total smokers included in the study (N=725), n	Messaged the TTS at least once during the 6-month period (n=245), n (%)	Did not message the TTS even once during the 6-month period, (n=480), n (%)	*P* value
Overall		725	245 (33.8)	480 (66.2)	N/A^a^
**Patient sex**					**.28**
	Female	469	165 (35.2)	304 (64.8)	N/A
	Male	256	80 (31.2)	176 (68.8)	N/A
**Patient age (years)**					**.05^b^**
	19-34	151	37 (24.5)	114 (75.5)	N/A
	35-55	361	127 (35.2)	234 (64.8)	N/A
	55-64	167	63 (37.7)	104 (62.3)	N/A
	≥65	46	18 (39.1)	28 (60.9)	N/A
**Patient race**					**.06**
	White	605	195 (32.2)	410 (67.8)	N/A
	Black or African American	60	28 (46.7)	32 (53.3)	N/A
	Other	22	9 (40.9)	13 (59.1)	N/A
**Patient education**					**.09^b^**
	Some high school and high school graduate	245	71 (29.0)	174 (71.0)	N/A
	Some college	306	109 (35.6)	197 (64.4)	N/A
	College graduate or more	166	64 (38.5)	102 (61.5)	N/A
**Readiness to quit**					**.96^b^**
	Not thinking of quitting and thinking of quitting	518	176 (34.0)	342 (66.0)	N/A
	Set a quit date and already quit	202	69 (34.2)	133 (65.8)	N/A
**Allow smoking at home**					**.04**
	No	408	125 (30.6)	283 (69.4)	N/A
	Yes	317	120 (37.8)	197 (62.2)	N/A
**Number of cigarettes smoked per day**					**.67^b^**
	0-10	218	72 (33.0)	146 (67.0)	N/A
	11-20	355	117 (32.9)	238 (67.1)	N/A
	>20	152	56 (36.8)	96 (63.2)	N/A
**Visited other smoking cessation websites before the study**					**.04**
	No	565	180 (31.8)	385 (68.2)	N/A
	Yes	160	65 (40.6)	95 (59.4)	N/A
**Quit attempt (1 day or more) in the past 12 months**					**.70**
	No	321	106 (33.0)	215 (67.0)	N/A
	Yes	404	139 (34.4)	265 (65.6)	N/A

^a^N/A: not applicable.

^b^Test for trend analysis.

### Message Use Frequency and Volume

A total of 1082 messages (TTSs: 565/1082, 52.2%; smokers: 517/1082, 47.8%) were exchanged between the smokers and TTSs. Among those smokers who messaged at least once, the mean number of messages sent was 2.6 (SD 4.5, median 1, IQR 1). The frequency of messages sent by TTS 1 was 341 (mean 6.1, SD 6.4; median 4, IQR 6), by TTS 2 was 370 (mean 9.7, SD 12.2; median 3, IQR 11), and by TTS 3 was 371 (mean 6.7, SD 8.9; median 2, IQR 7).

### Comparison of Tobacco Treatment Specialist Motivational Messaging Codes

#### Motivational Interviewing Self-Evaluation Checklist

The majority of motivational interviewing codes were those that supported client strengths (280/517, 54.1%) and promoted engagement (280/5170, 54.1%; Table 2). For example, a TTS message highlighting the client strength was **“**well it sounds like you certainly have good experience with nicotine replacement, which is good because you have an idea of what works and what doesn’t.”

**Table 2:** Frequency of codes in messages (N=517).

Scale, category of codes, and codes				Messages, n (%)
**Motivational Interviewing Scale**				
	**Supporting client strengths**			**280 (54.1)**
		Explored previous successes	N/A^a^	
		Explored positive qualities	N/A	
		Accentuated *any* motivation for change	N/A	
		Highlighted any efforts towards change	N/A	
		Used affirmations to highlight strengths, motivation	N/A	
	**Engagement**			**280 (54.1)**
		Worked to fully understand the problem and the client’s perspective before moving toward change	N/A	
		Focused on engagement before change	N/A	
		Used reflective listening to convey empathy and understanding	N/A	
		Used affirmations to build a positive relationship	N/A	
	**Eliciting change talk**			**110 (21.3)**
		Asked about concerns using open-ended questions or reflective listening	N/A	
		Asked for elaboration about concerns	N/A	
		Explored client values as they relate to change	N/A	
		Selectively responded to change talk with curiosity, interest	N/A	
		Explored pros and cons or decisional balance	N/A	
		Used *low-threshold* questions	N/A	
	**Promoting internal motivation**			**106 (20.5)**
		Elicited and respected the client’s goals for treatment	N/A	
		Explored values underlying the motivation for change	N/A	
		Supported autonomy in decision making	N/A	
	**Assessing motivation**			**78 (15.1)**
		Identified a target behavior	N/A	
		Identified stage of change	N/A	
		Used importance, confidence, and readiness ruler	N/A	
		Differentiated between different areas of motivation (eg, substance use vs mental health; treatment vs change)	N/A	
	**Addressing ambivalence**			**72 (13.9)**
		Normalized ambivalence	N/A	
		Explored ambivalence	N/A	
		Reframed ambivalence	N/A	
		Used a decisional balance	N/A	
		Avoided direct persuasion	N/A	
		Explored pros and cons of change	N/A	
	**Rolling with resistance**			**48 (9.3)**
		Affirmation (external reframe)	N/A	
		Empathic response	N/A	
		Reflective listening	N/A	
		Providing choice	N/A	
		Nondefensive response	N/A	
**Smoking Cessation Counseling Scale**				
	**Basic counseling**			
		If willing to quit, provide assistance	247 (47.8)	
		Ask if willing to quit	26 (5.0)	
		If not quitting, help identify barriers	23 (4.4)	
	**Advanced counseling**			
		Helping smokers prepare to quit^b^	206 (39.8)	
		Helping smokers anticipate barriers^c^	197 (38.1)	
		Managing relapses^d^	73 (14.1)	
		Advising smokers to set a quit date	47 (9.2)	
		Getting social support^e^	46 (8.9)	

^a^N/A: not applicable.

^b^Helping smokers prepare to quit codes include identify reasons and benefits for quitting; provide information for follow-up visits with doctor; and recommend over-the-counter nicotine patch, other medications.

^c^Help smokers anticipate barriers codes include anticipate challenges in the beginning; anticipate nicotine withdrawal; alcohol is associated with relapse; and review past quit attempts, what helped, and what led to relapse.

^d^Manage relapses codes include if relapse occurs, review and learn from experience; if relapse occurs, repeat quit attempts; if relapse occurs, reassess problems; and total abstinence is essential.

^e^Getting social support codes include get support from family, friends, and coworkers; and other smokers in house are not helpful.

#### Smoking Cessation Counseling Scale

SCC Scale code analysis showed that the TTS messages provided assistance to smokers if they were willing to quit (247/517, 47.8%; Table 2). For example, a TTS message that included this code was *When you feel you are ready, I am here to help you.* The TTS messages also included the use of advanced counseling strategies to help the smokers prepare to quit (206/517, 39.8%) and anticipate barriers (197/517, 38.1%), such as *Have you thought about ways other than smoking that can help you deal with stress after you quit? Perhaps this is an important first step. It helps to plan as much as possible before you quit to avoid slips*.

### Topical Content Codes: Comparison of Tobacco Treatment Specialist and Smoker Messages

**Figure 1:**
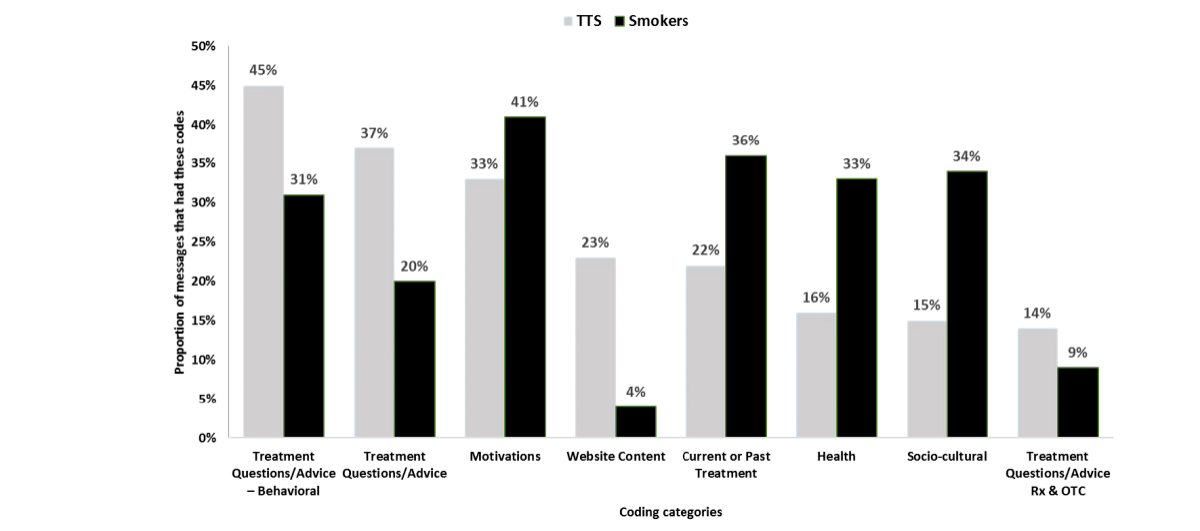
Proportion of topical content in messages: Smoker and tobacco treatment specialist messages. TTS: tobacco treatment specialist.

TTS messages and smoker messages differed in their content (Figure 1). The majority of smoker messages discussed motivations to quit (234/565, 41.4%) and current and past treatments, including their previous use of nicotine replacement therapy and medications (201/565, 35.6%). An example smoker message is as follows:

I just lost my mom and step father, both from smoking related disease's. I don't want to die like that ,so i will use all help, and support i can get.

The majority of TTS messages used behavioral strategies (233/517, 45.1%), offered advice on treatments (189/517, 36.5%), and highlighted motivations to quit (171/517, 33.1%). In the following example, a TTS response established the importance of general behavioral strategy:

Most importantly, you should ask your husband to not smoke around you. Your friends and family can be a big help during your quit attempt if you let them know what you need and they support you in quitting. Talk to them about what you will be experiencing during your quit attempt. For example, you may find you have a shorter temper during your quit process. Let your friends or family know this and not to take it personal if you are grumpy.

### Association Between the Point Prevalence Cessation Rate Assessed at 6 Months Using a 30-Day Window and the Selected Tobacco Treatment Specialist, the Content of Tobacco Treatment Specialist Motivational Messages, and Amount of Tobacco Treatment Specialist Use

After adjusting for gender, age, race, education, readiness at baseline, and number of cigarettes smoked per day at baseline, compared with TTS 1, the odds of quitting were lower for TTS 2 (OR 0.4; 95% CI 0.1-1.3) and TTS 3 (OR 0.9 95% CI 0.4-2.4), but not significant. There was no association between the codes in the TTS messages and smoking cessation (Table 3). There was also no association between the total SCC score and smoking cessation (OR 1.02; 95% CI 0.96-1.08).

The amount of TTS use (ie, the total number of messages sent to the TTS by each smoker) ranged from 0 to 42. In all, 66.2% (480/725) of smokers had never used the TTS, 26.1% (189/725) of smokers had used the TTS one or two times, and 7.7% (56/725) of smokers had used it more than two times. There was no association between the amount of TTS use and cessation. In the multivariable model after adjusting for gender, age, race, education, readiness at baseline, number of cigarettes smoked per day at baseline, and the selected TTS, compared with smokers who did not message the TTS, smokers messaging the TTS one or two times had a smoking cessation OR of 0.8 (95% CI 0.4-1.4), and those that messaged the TTS more than two times had a smoking cessation OR of 1.0 (95% CI 0.4-2.3; *P*=.50 for trend across categories). In the inverse probability weighted model, the smokers messaging the TTS one or two times had a smoking cessation OR of 0.7 (95% CI 0.4-1.3), and those that messaged the TTS more than two times had a smoking cessation OR of 0.7 (95% CI 0.3-1.7; *P*=.20 for trend across categories).

**Table 3:** Association between smoking cessation and the frequency of codes.

Scale, category of codes, and codes				Odds ratio (95% CI) using separate logistic regression models for each code
**Motivational Interviewing Scale**				
	**Supporting client strengths**			**1.12 (0.98-1.32)**
		Explored previous successes	N/A^a^	
		Explored positive qualities	N/A	
		Accentuated *any* motivation for change	N/A	
		Highlighted any efforts towards change	N/A	
		Used affirmations to highlight strengths, motivation	N/A	
	**Engagement**			**1.06 (0.88-1.27)**
		Worked to fully understand the problem and the client’s perspective before moving toward change	N/A	
		Focused on engagement before change	N/A	
		Used reflective listening to convey empathy and understanding	N/A	
		Used affirmations to build a positive relationship	N/A	
	**Eliciting change talk**			1.10 (0.74-1.62)
		Asked about concerns using open-ended questions or reflective listening	N/A	
		Asked for elaboration about concerns	N/A	
		Explored client values as they relate to change	N/A	
		Selectively responded to change talk with curiosity, interest	N/A	
		Explored pros and cons or decisional balance	N/A	
		Used *low-threshold* questions	N/A	
	**Promoting internal motivation**			**1.20 (0.74-1.82)**
		Elicited and respected the client’s goals for treatment	N/A	
		Explored values underlying the motivation for change	N/A	
		Supported autonomy in decision making	N/A	
	**Assessing motivation**			**1.05 (0.52-2.09)**
		Identified a target behavior	N/A	
		Identified stage of change	N/A	
		Used importance, confidence, and readiness ruler	N/A	
		Differentiated between different areas of motivation (eg, substance use vs mental health; treatment vs change)	N/A	
	**Addressing ambivalence**			**0.94 (0.55-1.63)**
		Normalized ambivalence	N/A	
		Explored ambivalence	N/A	
		Reframed ambivalence	N/A	
		Used a decisional balance	N/A	
		Avoided direct persuasion	N/A	
		Explored pros and cons of change	N/A	
	**Rolling with resistance**			**0.82 (0.35-1.92)**
		Affirmation (external reframe)	N/A	
		Empathic response	N/A	
		Reflective listening	N/A	
		Providing choice	N/A	
		Nondefensive response	N/A	
**Smoking Cessation Counseling Scale**				
	**Basic counseling**			
		If willing to quit, provide assistance	1.0 (0.69-1.42)	
		Ask if willing to quit	0.72 (0.18-2.97)	
		If not quitting, help identify barriers	1.44 (0.60-3.41)	
	**Advanced counseling**			
		Helping smokers prepare to quit^b^	1.0 (0.82-1.21)	
		Helping smokers anticipate barriers^c^	1.07 (0.88-1.31)	
		Managing relapses^d^	0.88 (0.54-1.45)	
		Advising smokers to set a quit date	1.24 (0.66-2.35)	
		Getting social support^e^	1.04 (0.6-1.80)	

^a^N/A: not applicable.

^b^Helping smokers prepare to quit codes include identify reasons and benefits for quitting; provide information for follow-up visits with doctor; and recommend over-the-counter nicotine patch, other medications.

^c^Help smokers anticipate barriers codes include anticipate challenges in the beginning; anticipate nicotine withdrawal; alcohol is associated with relapse; and review past quit attempts, what helped, and what led to relapse.

^d^Manage relapses codes include if relapse occurs, review and learn from experience; if relapse occurs, repeat quit attempts; if relapse occurs, reassess problems; and total abstinence is essential.

^e^Getting social support codes include get support from family, friends, and coworkers; and other smokers in house are not helpful.

## Discussion

### Principal Findings

Our discussion focuses on three topics: (1) approximately, one-third of the smokers who were offered an opportunity to asynchronously communicate with the TTS over the web chose to use the service, suggesting some level of interest in the service; (2) the coding analysis demonstrated that the TTSs were able to deliver best practice–based counseling on this asynchronous medium; and (3) the finding of no association between the amount of TTS use and smoking cessation has important implications.

Previous interventions with web-based counseling for a variety of behavioral and mental health conditions have had inconsistent results, with negative studies being a common finding. However, as these studies did not explore the message content, we cannot say whether these interventions delivered counseling that included evidence-based communication patterns. Thus, one goal was to understand the content of the messages. In our previous publication, we have qualitatively analyzed the communication between the smoker and the TTS and identified seven basic themes in the communication—talk about the process of quitting, barriers to quitting, reasons to quit, quit history, support and strategies for quitting, quitting with medication, and quit progress [15]. This paper adds to this evaluation by conducting a semiquantitative coding analysis and evaluating the association between the codes and the number of messages and smoking cessation. We also evaluated whether the content and volume of messages were related to the cessation outcomes at 6 months.

As noted, a third of our participants chose to message the TTS without actively prompting them. Please note that we did not require the smoker to message the TTS. In a study with smokers from Arkansas Children’s Hospital, 21% of smokers participated following a fax referral [38]. Fax referral is a process in which smokers are referred to a quit line by simply faxing a referral form to the quit line. Quitline TTS, then, proactively calls the smoker to setup the counseling sessions. In an observational study of faxed referrals to the Ohio Tobacco Quit Line, 23% (n=1616) of smokers were able to be enrolled out of a total of 6951 faxed referrals [39]. These results may suggest that there is some merit to our assumption that asynchronous counseling offers a lower barrier to access, acknowledging that the participants in our study may be more motivated to quit than the general population of smokers, as indicated by their registration on WATI. Furthermore, among those who communicated with the TTS, the majority (32/45, 71%) indicated the communication was helpful.

We used the Motivational Interviewing Self-Evaluation Checklist and the SCC Scale for developing our codes to evaluate the TTS messaging. As noted above, the Motivational Interviewing Self-Evaluation Checklist was originally developed as a self-evaluation tool to improve motivational interviewing skills [28-32], while the SCC Scale was originally developed to help in assessing, improving, or testing evidence-based methodologies for SCC [33]. A contribution of our work is adapting these to code messages between TTSs and smokers on the web. Some of the messages included motivational interviewing and other behavioral strategies. The techniques used included supporting client strengths and promoting engagement, among others. TTSs were able to include messaging to help smokers prepare to quit and anticipate barriers.

In our content analysis of the smokers’ messages, we found that smokers’ messages most often discussed motivations to quit and current and past treatments used for smoking cessation, including their previous use of nicotine replacement therapy and medications. This suggested that smokers were able to express their motivations to quit, as well as the challenges they face while quitting. In addition, smokers were also able to express what worked or did not work in their past treatment. This ability of the smokers to include these concepts in their messages is important as it allows the TTSs to tailor their messaging to the smokers. This was evident in our analysis of the TTS messaging, during which TTSs were able to incorporate behavioral strategies, offer advice on treatments, and highlight motivations to quit.

We did not find any association between smoking cessation and the codes in the TTS messages, the selected TTS, and the amount of TTS use. As noted above, only a third of our participants chose to message the TTS and even among these participants, the mean number of messages sent was 2.6. This may have contributed to our inability to detect an effect. The tobacco treatment guidelines recommend four or more sessions to increase the effectiveness of counseling [1]. A previous meta-analysis has also indicated that the session length is an important factor in the effectiveness of counseling [1]. For the light-touch, low-intensity approach of asynchronous counseling, the number of interactions may need to be higher. In future work, we plan to explore approaches to increase engagement, both in terms of overall use of asynchronous counseling and the number of interactions between TTSs and smokers. First, accessing the TTS by logging into the web interface may be a barrier. Future studies could test whether text messaging and mobile phone–based access further reduce barriers to access. Second, users may need tailored and proactive messaging to continually motivate and encourage them to participate in asynchronous counseling. As noted, our study identified certain characteristics that were significantly associated with participation; compared with those who did not message the TTS, smokers who messaged the TTS were those who allowed smoking at home and who had visited a WATI before this study. These characteristics could potentially be used to tailor messages to motivate the smokers to engage in asynchronous counseling with the TTS.

Future trials should test asynchronous counseling in combination with synchronous counseling, as asynchronous counseling may be most beneficial when used as an augmentation to synchronous counseling. For example, the asynchronous counseling could be used in between synchronous counseling sessions to continually support the smoker. The timing (whether the synchronous counseling session precedes or follows the start of the asynchronous counseling) and the number of synchronous counseling sessions need to be tested. Furthermore, it is difficult to find an overall impact of TTS use because the content of the messages varied considerably.

### Limitations

This was an observational study. Furthermore, not all smokers who participated in the larger randomized clinical trials participated in this study. Although this circumstance helped identify those who are likely to use the service, this may have caused some selection bias. The number of interactions between the TTSs and the smokers were also low. As this was a secondary, exploratory analysis, our study had limited power. Comparing TTS use=0 with TTS use=top quartile, we found a difference in cessation of 3% (top quartile: 9/56, 16%; 0 use: 62/480, 12.9%). With the sample available, we were powered to detect a difference of 11%.

### Conclusions

Asynchronous counseling may be able to augment synchronous counseling, providing smokers the ability to connect with counselors at their convenience. We did not find an association between asynchronous counseling and smoking cessation. There are several potential explanations for this finding, including a lack of intervention engagement by participants, a lack of intervention fidelity (the counselors did not deliver evidence-based support), and a lack of power. We have now demonstrated that evidence-based cessation counseling did occur. Our discussion outlines several approaches to potentially increasing the effectiveness of asynchronous counseling, including a discussion of approaches to increase participant engagement, and testing the asynchronous counseling in combination with synchronous counseling. Future studies are needed to address the best ways to use asynchronous counseling for helping smokers.

## Appendix

Multimedia Appendix 1 Decide2Quit website.

Multimedia Appendix 2 Topical content coding schema.

Multimedia Appendix 3 Email communications between smokers and TTS.

## Abbreviations

IRR: interrater reliability
OR: odds ratio
SCC: smoking cessation counseling
TTS: tobacco treatment specialist
WATI: web-assisted tobacco intervention
