# Supplementary figures and images for: Secure Asynchronous Communication Between Smokers and Tobacco Treatment Specialists: Secondary Analysis of a Web-Assisted Tobacco Intervention in the QUIT-PRIMO and National Dental PBRN Networks
Source: J Med Internet Res. 2020 May 6;22(5):e13289. doi: 10.2196/13289 (PMC7240437; doi:10.2196/13289)

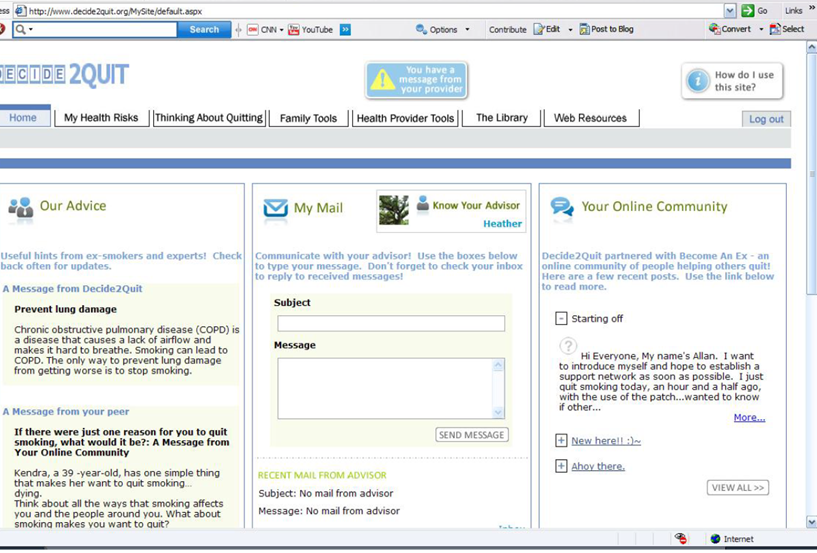


TTS Column

Admin Interface (TTS View)


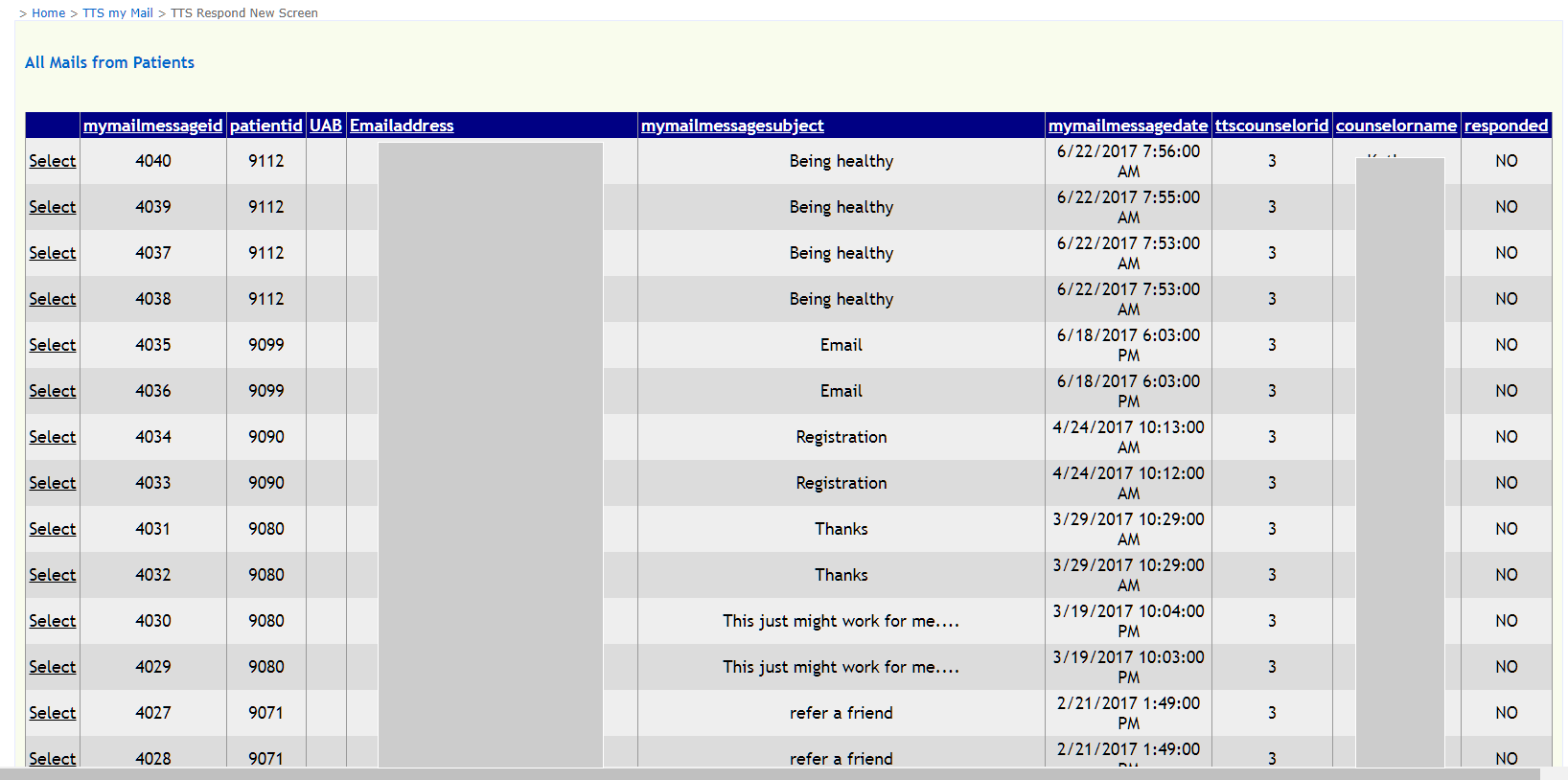


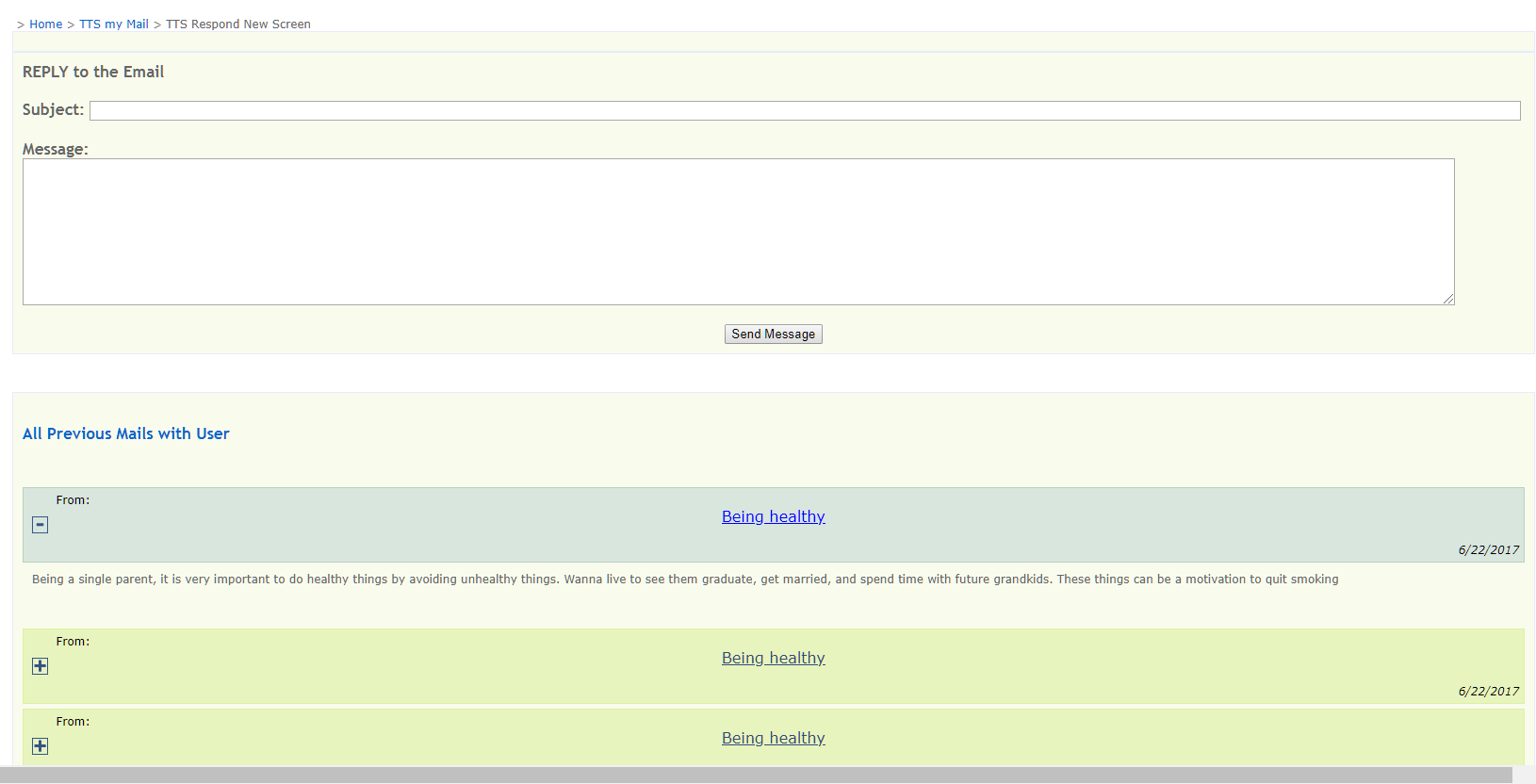

Supplement: Multimedia Appendix 1 [file jmir_v22i5e13289_app1.doc]
