# Supplementary material for: Secure Asynchronous Communication Between Smokers and Tobacco Treatment Specialists: Secondary Analysis of a Web-Assisted Tobacco Intervention in the QUIT-PRIMO and National Dental PBRN Networks
Source: J Med Internet Res. 2020 May 6;22(5):e13289. doi: 10.2196/13289 (PMC7240437; doi:10.2196/13289)
Supplement: Multimedia Appendix 2 [file jmir_v22i5e13289_app2.doc]

| **Coding Categories (TTS)** | **Code Definitions** | **Messages** |  |  |  |  |
| --- | --- | --- | --- | --- | --- | --- |
|  |  |  |  |
|  |  |  |  |
| ***Motivational Interviewing Content*** | | |  |  |  |  |
| **Supporting client strength** | Explore previous successes; Explore positive qualities; Accentuate *any* motivation for change, Highlight any efforts towards change, Use affirmations to highlight strengths, motivation | **TTS:** You are making tremendous progress |  |  |  |  |
| **Engagement** | Ask questions to fully understand problem and patient’s perspective before moving towards change, Use reflective listening/summaries to convey empathy and understanding, Use affirmations to build a positive relationship | **TTS:** Having the support of family and friends in a quit attempt is important. It sounds like you have many people willing to help you make this a success. You're definitely on the road to quitting. Let me know if you have any questions related to your quit attempt |  |  |  |  |
| **Eliciting change talk** | Ask about concerns using open-ended questions, Ask for elaboration about concerns, Explore patient’s values as they relate to change, Respond to positive change talk with curiosity & interest | **TTS:** Can you tell me a little more about what situations are making it difficult for you to stay quit? |
| **Promoting internal motivation** | Elicit and respect goals for treatment, Explore values underlying motivation for change, Support decision making | **TTS**: It sounds like you have very good motivation to quit |  |  |  |  |
| **Assessing motivation** | Clarify patient’s stage of change; Discuss importance & confidence to reach targeted behavior; Discover plans or intentions; Ask about motivations | **TTS**: That is the challenge of quitting—knowing how to cope without a cigarette. |  |  |  |  |
| **Addressing ambivalence** | Normalize or explore ambivalence; Explore pros and cons of change; Discuss barriers, Assist patients to recognize ambivalence | **TTS:** In regards to weight gain, try to switch to more healthy snacks |  |  |  |  |
| **Rolling with resistance** | Respond to resistance by avoiding confrontation/direct persuasion; Meet resistance with: reframing, affirmation, empathic response, reflective listening, providing choice, non-defensive response | **TTS**: You may want to consider other energy-boosting activities |  |  |  |  |
| ***Smoking Cessation Counseling Content*** | | |  |  |  |  |
| **Basic counseling** | | |  |  |  |  |
| **If willing to quit provide assistance** | If tobacco users are willing to quit, I provide resources and assistance | **TTS:** I can help you think through the benefits of quitting and things you can do to lessen the urges |  |  |  |  |
| **Ask if willing to quit** | I ask tobacco users if they are willing to quit | **TTS:** Have you ever quit smoking before, even for a short period of time? |
| **If not quitting, help identify barriers** | If tobacco users are not willing to quit, I provide resources and help patient identify barriers to quitting | **TTS:** What would you say is your biggest barrier to quitting now? There is also more information about figuring out your barriers to quitting on the website. |  |  |  |  |
| **Advanced counseling** | | |  |  |  |  |
|  |  |  |  |
| **Help anticipate challenges in the beginning** | I help the patient anticipate challenges, particularly during the critical first few weeks | **TTS**: The most important thing is getting through the initial phase when your urge to smoke is the strongest. |  |  |  |  |
| **Help patients anticipate nicotine withdraw** | I help patients anticipate nicotine withdraw | **TTS:** The longer you stay quit, the easier it will be to control cravings and the lesser the urge to smoke will be. It's also good that you are taking it one day at a time |
| **Advice patients if relapse occurs, review and learn from experience** | I advise patients that if relapse occurs, they should reassess the pharmacotherapy use and problems | **TTS**: Think about those 2 days you were able to quit. I'm sure you learned some things about yourself that will help you this time |
| **Advice if relapse occurs, reassess problems** | I advise patients if relapse occurs, they should review the circumstances and learn from the experience | **TTS:** |  |  |  |  |
| **Advice if relapse occurs, repeat quit attempts** | I advise patients if relapse occurs, they should repeat the quit attempt– it is part of the quitting process | **TTS:** Whatever your plan is, be prepared for when the urge hits |  |  |  |  |
| **Advice that other smokers in house aren't helpful** | I advise patients that having other smokers in household hinders successful quitting | **TTS:** Avoid being around your friend when you know he or she will be smoking |  |  |  |  |
| **Advice that alcohol is associated with relapse** | I advise patients that drinking alcohol is strongly associated with relapse | **TTS:** |  |  |  |  |
| **Advice that total abstinence is essential** | I advise patients that total abstinence is essential– not even a single puff | **TTS:** One smoke is not worth the pain of having to start over |  |  |  |  |
| **Advice to get support from family , friends coworkers** | I advise smokers to get support from family, friends, and coworkers | **TTS:** Having the support of those around you is very important when quitting smoking |  |  |  |  |
| **Advice smokers to set a quit date** | I advise smokers to set a quit date | **TTS:** It may be best to begin by setting a quit date, or deciding what day you want to quit. |  |  |  |  |
| **Identify reasons and benefits for quitting** | I identify reasons for quitting and benefits of quitting | **TTS:** What are some reasons you want to quit? Family? Health? |  |  |  |  |
| **Provide information for f/u visits with doctor** | I provide information for follow-up visits with the patient’s doctor | **TTS:** I highly recommend you talk with your doctor |  |  |  |  |
| **Recommend OTC nicotine patch, other medications** | I recommend use of over-the-counter nicotine patch, gum, or lozenge; or get a prescription for nasal spray, inhaler, or buproprion SR unless contraindicated | **TTS:** Nicotine replacement would be helpful to you- like the patch or gum. These are available over the counter and can really help with the cravings. |  |  |  |  |
| **Review past quit attempts, what helped & what led to relapse** | I review past quit attempts– what helped, what led to relapse | **TTS**: Your previous quit of 6 months can be very helpful in your current quit attempt. Think back about what worked for you and what didn't. |  |  |  |  |
| ***Topical Contents*** | | |  |  |  |  |
|  |  |  |  |
| **Motivations** | | |  | | | |
|  | | | |
| **Smoking motivations** | Why someone currently smokes, what drives them to smoke | **TTS:** What do you like about smoking?  **Smoker:** I enjoy it too much. It's my only vice. How do I even get to the point of thinking strongly about it |  | | | |
| **Quit motivations** | Why someone wants to quit smoking | **TTS:** Good reasons to quit smoking- your lung cancer scare and money. You are definitely making progress to being a nonsmoker!  **Smoker:** I want to quit for myself and my children and grandchildren. |  | | | |
| **Current or past treatment** | | |  | | | |
| **Current /past treatment Rx or NRT** | Current or previous use of prescription medication or nicotine replacement therapy to treat smoking addiction, NOT questions or advice | **TTS: You are using the patch**  **Smoker: Ent off patch during day and put 1/2 of #2 patch on late afternoon when craving begins. Also tried 2 milligram Commit instead of patch** |  | | | |
| **Current/past behavioral** | Current or previous use of any behavioral strategy (see behavioral strategy definitions) to treat smoking addiction, NOT questions or advice | **TTS: Is there something you can do BEFORE having the temptation to smoke after eating that will make you feel the urge less**  **Smoker: Get rid of the one pack of cigarettes that I have in an out-of-the way place on my porch. I have to mentally decide to take that final step** |  | | | |
| **Current/past side effects** | Current or previous side effects from smoking cession prescription medication or nicotine replacement therapy, NOT questions or advice | **TTS: You can handle the side effects of Chantix.**  **Smoker: When I put them on my arm, they make my arm sore** |  | | | |
| **Current/past other** | Any other current or previous treatment for smoking addiction that isn’t Rx, NRT or behavioral, NOT questions or advice | **TTS: Get enough sleep - this will improve your mood, immune function, and ability to cope with stress.**  **Smoker:** |  | | | |
| **Questions current/past smoking treatment** | QUESTIONS about past smoking treatment, TTS gathering background info | **TTS: What else have you used to help you quit before?**  **Smoker: I have been praying to stop or reduce smoking** |  | | | |
| ***Social cultural*** | | |  | | | |
| **Social support** | | |  | | | |
| **Positive social support** | Friends or family don’t provide emotional support for d2q user, DO NOT code family general but may overlap with family problems | **TTS:** You are fortunate to have the support of your husband.  **Smoker**: My FB people have been very supportive too |  | | | |
|  | | | |
| **Negative social support** | Friends or family provide emotional support for d2q user, DO NOT code family general but may overlap with family problems | **TTS:** Quitting smoking is hard work and it can be even harder when you have to do it alone.  **Smoker:** Older daughter who lives with us not helpful at all |  | | | |
| **Family ( general concerns, problems, money)** | Comments about family that aren’t covered under social support, family problems, or responsibilities, family that directly or indirectly impact the user or any comment about costs, paying bills, income or saving money | **Smoker:** My husband and I are not working right now and I wondered if I could get any assistance from you  **TTS:** Have you looked at how much you spend on your cigarettes? Could this offset the cost of patches? Unfortunately these are the only options I know regarding the patches.  ------------------------------------------------------------------------------  **Smoker**: Unfortunately I couldn’t afford cigs, and was mostly bumming and now can definitely not afford the patch.  **TTS:** I don't know of any other source for free patches  -----------------------------------------------------------------------  **Smoker:** He used the patch. On Thursday he found two cigarettes he hadn't thrown out when we quit 7/11. Then he smoked them and he smoked again. |  | | | |
| **Personal history** | A short biography of the user, if ONLY focused on smoking treatment code as past/current treatment, not personal history. Past treatment and history codes may exist in the same sentence but don’t overlap them | **TTS**: Could I get a little more information about your smoking history first? Are you currently preparing to quit or in the middle of a quit attempt? Have you tried before?  **Smoker:** I am a smoker of over 30 years. I quit once for 6 years |  | | | |
|  | | | |
| **Health** | | |  | | | |
| **Weight gain** | Concern or discussion about weight problems | **TTS**: To have a plan that will help minimize any weight gain if you are worried about that  **Smoker**: I am afraid I will gain weight |  | | | |
| **Medical problems / Sleeping problem** | Medical concerns NOT directly related to weight gain or non-diagnosed sleep problems | **TTS:** Sorry to hear of your recent diagnosis.  **Smoker:** I have Diabetes and Fibromyalgia and a few other things wrong with me and in a lot of pain all day long. |  | | | |
| **Positive Emotions** | Happy, Excited or | **Smoker:** I am feeling better emotionally  **TTS:** So glad things are going well |  | | | |
|  | | | |
| **Negative emotions** | Tension/Stress, Sadness, Anger | **Smoker:** I am so weak and really down about it.  **TTS:** It is important to have other ways of dealing with stress, such as taking a walk or doing deep breathing exercises |  | | | |
|  | | | |
|  | | | |
| **Treatment Question/Advice - Behavioral** | | |  | | | |
| **Behavioral Strategies General** | Gives treatment advice or questions about using or avoiding specific behaviors to quit smoking cigarettes. NOT substitution or distraction | **TTS:** Removing things in your home or car that trigger you to smoke like ashtrays or cigarettes.  **Smoker:** Any suggestions for alternative ways to get over these stressful moments |  | | | |
| **Behavioral Strategies Substitution** | Gives treatment advice or has questions about Substituting something in the place of smoking/cigarettes, such as toothpicks or (regular) gum | **TTS**: There are a several behavioral things you can do to fight urges as well (instead of using the gum). Some people like to chew on straws or cinnamon flavored toothpicks when they have cravings  **Smoker**: I am getting ready to chew my first piece of Nicorette gum |  | | | |
| **Behavioral Strategies Distraction** | Gives treatment advice or has questions about doing an activity to help Distract from the smoking craving, such as playing a game or running | **TTS:** Try to make increasing physical activity a part of your daily activities (use the stairs instead of the elevator, park in farther parking spots for times when you do use your truck)  **Smoker:** I put exercise DVDs in when I want to smoke, and exercise. |  | | | |
| **Quit Date / Quit Plan** | Gives advice or has questions about the Date someone plans to quit smoking by/  Gives advice or has questions about the Plan someone has to quit smoking, may involve some of the above categories, how to cope without cigarettes | **TTS:** Have you thought about a new day when you'd like to quit smoking?  **Smoker:** My target quit day is Monday, August 30^th^.  -------------------------------------------------------------------------------  **TTS:** Another thing people do when they quit is to make a quit plan so that you decide when you want to quit and make preparations for that day  **Smoker**: I have a feeling that I am going to have to add something more to my quit plan to make it past this. Please send me any suggestions that you think might be helpful. |  | | | |
| **Treatment Question/Advice** | | |  | | | |
| **Smoking Counseling** | Gives treatment advice or has questions about in person smoking counseling | **TTS**: I'm glad to answer any questions you have and help you plan your quit attempt. Will this be your first time trying to quit smoking?  **Smoker:** what tips you have for individuals that desire to stop smoking |  | | | |
| **Quit line** | Gives treatment advice or has questions about Quit-lines | **TTS:** Each state has its own tobacco quit line (1-800-QUIT-NOW)  **Smoker:** I will contact that number,and get advice |  | | | |
| **Talk to doctor** | Patient told to speak to doctor about treatment, overlaps with SCC code | **TTS:** If you are interested in using these products I recommend checking with your doctor about what will work best for you.  **Smoker**: waiting to see my doctors |  | | | |
| **Relapse prevention** | Gives treatment advice or has questions about what can be done to avoid relapse and be preparing for the difficulty of staying quit | **TTS:** Each time you resist, you are learning new ways of coping that will help you in the long run. Don't give up  **Smoker:** I need to figure out how to not go back |  | | | |
| **Q/A about Smokeless Tobacco or other future treatments** | Gives treatment advice or has questions about smokeless tobacco or other questions or advice about future treatments to stop smoking | **TTS:** smokeless tobacco user  **Smoker:** smokeless tobacco  --------------------------------------------------------------------------------  T**TS**: Please let me know if you have any specific questions about your quit attempt  **Smoker**: How to rid the urge or desire without depending on any medications |  | | | |
|  | | | |
| **Treatment Question/Advice - Rx & OTC** | | |  | | | |
|  | | | |
| **Nicotine Replacement Therapy or Prescription Drugs** | Gives treatment advice or has questions about NRT or prescription drugs | **TTS:** Have you ever tried nicotine replacement (such as the patch or gum)  **Smoker:** got the patches here but I’m afraid to use them, I don’t know if they will make me feel funny.  -------------------------------------------------------------------------------  **TTS:** Chantix has been found to be helpful for people who want to quit smoking. However, as with all drugs, it is important to weigh the benefits with the risks.  **Smoker:** My doctor prescribed Chantix to help me quit smoking |  | | | |
|  | | | |
| **Electronic Cigarettes** | Gives treatment advice or has questions about electronic cigarettes | **TTS:** Also, mention to them you are going to use the e-cig. This has not been approved by the FDA and we can't recommend you use this so please get their opinion before starting  **Smoker:** I ordered a electronic cigeratte to see if that helps me quit |  | | | |
| **Website Content** | | |  | | | |
| **Technical problems** | User having trouble with messaging or website | **TTS:** I'm sorry you've not been able to find your emails from your doctor. They should appear on the top of the page in a blue triangle with a yellow exclamation mark. Do you see anything like that? Have you sent me emails previously? This is the first one I've received from you.  **Smoker:** When I hit, "send message," I got a note that said something went wrong and that I needed to navigate back to the home page. I want to try to send this and see that it goes through before I rewrite my original message. |  | | | |
| **Questions D2Q content** | User wants clarification of something on the website, non-technical | **TTS:** How to make the website more effective for you.  **Smoker:** Can I still use this site to help me |  | | | |
| **Refer to D2Q section or outside website** | TTS refers back to D2Q website or TTS refers to outside website | **TTS:** There is more information about barriers to quitting on the website. From the home page of www.decide2quit.org, click on “Thinking About Quitting” and then click on “What will I need to overcome calculator”  ---------------------------------------------------------------------------------  **TTS:** Check out this resource from the U.S. Department of Health and Human Services that gives a play-by-play of what happens when you quit smoking: <http://www.womenshealth.gov/quit-smoking/tools/calendar.cfm> |  | | | |
| **Feedback from patients** | | |  | | | |
|  | | | |
|  | | | |
|  | | | |
| **Feedback on TTS (positive or negative)** | Users complimenting or give negative remarks to TTS counselors about their messages, NOT the website or technical | **Smoker:** Thank you for being here and for all that you do  **Smoker:** The last message was incredibly long, it was way too involved and suggestive, it also didn't sound like the counselor I had been receiving messages from before |  | | | |
|  | | | |
| **Feedback on website (positive or negative)** | Users saying they like or dislike the website, found it or Not helpful/informative/interesting, NOT specifically related to TTS messages | **Smoker**: Great site, by the way! |  | | | |
