# Supplementary material for: Secure Asynchronous Communication Between Smokers and Tobacco Treatment Specialists: Secondary Analysis of a Web-Assisted Tobacco Intervention in the QUIT-PRIMO and National Dental PBRN Networks
Source: J Med Internet Res. 2020 May 6;22(5):e13289. doi: 10.2196/13289 (PMC7240437; doi:10.2196/13289)
Supplement: Multimedia Appendix 3 [file jmir_v22i5e13289_app3.doc]

| **Messages** | **TTS/**  **Smoker** | **Smoking status** | | **Codes** |
| --- | --- | --- | --- | --- |
|  |  | **30 days** | **7 days** |  |
| Single African American woman, 57 yrs. old have smoked since age 21. I have many illnesses now: fibromyalgia, osteoarthritis in hips are the main ones that constantly leaves me in pain, also I was diagnosed with chronic back pain. Finally decided to quit smoking to help alleviate some of this pain. I was smoking at least 1 pack daily, but when I get upset of course I would smoke more. I am also lying on my stomach because of lower back pain, also unable to sit for long periods of time. I pray every day since I quite, to keep me strong and positive thinking. | **Smoker 1**  1211 | -- | NO | **Contenttreatmentother**  **contenthealthmedicalproblems**  **contentsocioculturalpersonalhist**  **contentmotivationssmokingmotivat**  **contentmotivationsquitmotivation** |
| Hi. That's great you have quit! You have a significant smoking history...what are some of the tools you are using to help with cravings? Have you identified any specific difficult situation or circumstances in which it is more difficult to fight the cravings? How long have you been quit? Please let me know if you have any questions. Take a look around the site, perhaps there is something new that can help you! Take care, Kathryn Certified Tobacco Treatment Specialist | TTS  1217 |  |  | **misupportingclientstrengths**  **miengagement**  **sccbcifwillingtoquitprovideassis**  **sccachelpanticipatechallengesinb**  **contenttreatmentpastsmoking**  **contentmotivationssmokingmotivat**  **contentwebrefertod2qsection** |
| I started using nicotine patches on 6/24, I was doing so good I surprised myself. There have been many attempts in past, never got passed the 3rd day. On Saturday evening I received a call, my best friends’ boyfriend had just died. It was such a shock, I instantly asked my son for a cigarette. I have not used a patch afterwards. I told my son that I must start using the patch again and stick to it. | **Smoker 1**  1223 |  |  | **Contenttreatmentrxornrt**  **contentmotivationssmokingmotivat** |
| Hi. Many smokers slip while quitting smoking! The important thing to remember is to not look at it as a failure, but learn from your slip. For instance, dealing with the shock of a loss and turning to cigarettes as a reliable coping mechanism is completely understandable, but what if you planned ahead and found other activities or things to do when faced with a difficult situation. You might even run into a difficult period of cravings....what sorts of things do you have in place to deal with cravings? Let me know if you need help brainstorming.... Take Care, Kathryn | TTS  1226 |  |  | **Mielicitingchangetalk**  **miengagement**  **sccbcifwillingtoquitprovideassis**  **sccacadvicepatientsifrelapseoccu**  **contentmotivationssmokingmotivat**  **contenttxquesadvicerelapsepreven**  **contenttxquesadvbehavbehavstratg**  **contenttxquesadvbehavbehavstratd** |
| I am 2 weeks into not smoking. I always seem to stumble along the way when quitting. I have been trying to quit by myself for years now. My doctor referred me to this website, hopefully this website will help. | **Smoker 2**  1074 | NO | NO | **contenttreatmentother**  **contenttxquesadvicetalkdoctor** |
| Congratulations on quitting! I hope this website will help also, and please let me know if I can answer any questions along the way! Are you currently using any nicotine replacement therapy? Any medications? Quitting smoking can be very hard (as I'm sure you already know :-)), but you have made a great step in logging on to the website and in contacting me. Take a look around the site and let me know if you have questions. Take Care, Kathryn Certified Tobacco Treatment Specialist | TTS  1081 |  |  | **misupportingclientstrengths**  **miengagement**  **sccbcifwillingtoquitprovideassis**  **sccachelppatientsanticipatenicot**  **contenttreatmentpastsmoking**  **contentwebrefertod2qsection** |
| It’s been 7 days now that I've been off of Nicorette gum. I am proud of myself but my head is still dizzy at times. I’m sure it will pass. I've done all this before but this will be the last. No more just one cigarette urges | **Smoker 2**  1103 |  |  | **contenttreatmentrxornrt**  **contenttreatmentsideeffects**  **contenttreatmentother**  **contenthealthpositiveemotions** |
| Hi. It's great that you are still quit. Don't forget that the gum is there to help; it sounds like you had a goal to be done with the gum? Also, you sound very determined, and if you've had several quit attempts, I can understand why. May smokers have several quit attempts before being successful, and I believe a part of what helps them be successful does not want to deal with the frustration. The more attempts you have the more you know and learn about how you react and deal with triggers and cravings. Keep up the great work and let me know if you have questions. Take Care, Kathryn | TTS  1120 |  |  | **misupportingclientstrengths**  **miengagement**  **sccacrecommendotcnicotinepatchot**  **sccacreviewpastquitattemptswhath**  **contentmotivationsquitmotivation**  **contenttxquesadvbehavbehavstratg**  **contenttxquesadvrxotcnrt** |
| I am a 25 year old mother of 2 that is very interested in quitting smoking. I have a hard time not smoking in the car. What are some motivational tools that I can use to not smoke in the car. Bear in mind, I do know techniques and things that I should be doing, but what is one thing that you can recommend that can really trigger cessation and motivation. I at one time smoked 2 packs of cigarettes a day and have heavily curbed my intake. Most days I only smoke around 5 cigarettes, but am starting back to work today...which involves a lot of driving. Thanks for your input and help and will enjoy hearing your response. | **Smoker 3**  1059 | NO | NO | **contentfbpositivefeedbackontts**  **contenttreatmentother**  **contentsocioculturalpersonalhist**  **contentmotivationssmokingmotivat**  **contenttxquesadvbehavbehavstratg** |
| Hi. So, it sounds like we need to get hard at work brainstorming ideas so that we can come up with one that will work for you! It is great that you have already identified that smoking in the car is one (or maybe THE one) hardest for you to give up. Also, it is very good that you are down in number of cigarettes per day - this will really help you in the long run. You mentioned wanting ideas that will really trigger cessation and motivation - that will have to come from you! One task may be to get some kind of voice recorder and while driving - instead of continuous smoking, allow yourself one, and make it be after you complete the task of brainstorming (and recording this) all the reasons why YOU want to quit! You are young and you have young children....a really great motivation can be that kids of parents who quit while young are less likely to ever smoke themselves! What are some of your other motivations? Take Care, Kathryn Certified Tobacco Treatment Specialist | TTS  1068 |  |  | **misupportingclientstrengths**  **mipromotinginternalmotivation**  **miengagement**  **sccacidentifyreasonsbenefitsforq**  **sccachelpanticipatechallengesinb**  **contentsocioculturalfamilygenera**  **contentmotivationssmokingmotivat**  **contentmotivationsquitmotivation**  **contenttxquesadvbehavbehavstratg** |
| Thank you for being so positive in your response. I do like the voice recorder idea and will give that a shot. I do constantly think about smoking/not smoking and about ideas on how to not think about it so much. It is working some, but with returning to work after maternity leave I am also afraid that I will fall right back into a pack a day just out of habit. I do enjoy talking with someone like yourself that has successfully quit. My mother is also a smoker that has been quit for seven years now. She had quit for four years when I was younger, returned to smoking for two years, and then quit again for seven. She is also motivation for me to quit. I do not allow myself to smoke around her and it feels normal now at her home to not smoke at all. I do have the occasional craving but it quickly vanishes. This is also a goal that I hope to obtain for my home. We do not smoke in the house, but I enjoy sitting on my front porch and smoking. Sigh, so much to accomplish, but it can be done :) thanks again for your help. | **Smoker 3**  1073 | NO | NO | **contentfbpositivefeedbackontts**  **contenttreatmentbehavioral**  **contenthealthnegativeemotions**  **contentsocioculturalsocsuppos**  **contentsocioculturalfamilygenera**  **contentmotivationssmokingmotivat**  **contentmotivationsquitmotivation**  **contenttxquesadvbehavbehavstratg** |
| You are welcome! Believe it or not, someday it will be normal for you to not smoke - everywhere. It takes time, and over time it becomes easier and easier. Don't forget to not be too hard on yourself, as long as you keep your ultimate goal, and stay positive that you can do it! Let me know how it goes, and what questions you may have come up! Take Care, Kathryn | TTS  1080 |  |  | **miengagement**  **sccbcifwillingtoquitprovideassis** |
| I did set a quit date for last week and the day has come and gone and I am still smoking. I am now backing to smoking between a half pack and a pack/day. I feel really anxious when I know that I want to quit, and I crave them more. This is very frustrating and in turn makes me want to smoke when I am thinking about not smoking! My head congestion has increased, in part to allergies, but I can tell a big difference now that I am smoking more...please help with ways to lower the anxiety. | **Smoker 3**  1127 |  |  | **contenttreatmentbehavioral**  **contenttreatmentother**  **contenthealthmedicalproblems**  **contenthealthnegativeemotions**  **contentmotivationssmokingmotivat**  **contentmotivationsquitmotivation**  **contentmotivationsquitmotivation**  **contenttxquesadvbehavbehavstratg**  **contenttxquesadvbehavquitdate** |
| Hi. It is normal for a smoker to have anxiety about what life will be like without cigarettes. You may want to look into a relaxing technique or meditation to help you overcome your anxiety, and you should always talk to your doctor about any anxiety you may be experiencing. What are some of the thoughts you have when you turn to a cigarette? One good thing to begin with is to write down how you are feeling and what you are thinking when you reach for your cigarettes. Start with knowing how you use them - then you can maybe understand how you can lose them! Take Care, Kathryn | TTS  1132 |  |  | **mielicitingchangetalk**  **miaddressingambivalence**  **sccbcifnotquitinghelpidentifybar**  **sccacprovideinformationforfuvisi**  **contenthealthnegativeemotions**  **contentmotivationssmokingmotivat**  **contenttxquesadvicetalkdoctor**  **contenttxquesadvbehavbehavstratg** |
